# Serum Cystatin C and the Urinary NAG Index Identify an Altered Renal Biochemical Phenotype in Clinically Healthy Geriatric Dogs with Normal Serum Creatinine: A Three-Tier Cross-Sectional Study

**DOI:** 10.3390/vetsci13090881

**Published:** 2026-08-28

**Authors:** Andrei Răzvan Codea, Alexandra Biriș, Daniela Neagu, Alina Diana Haşaş, Cristian Popovici, Aurora Livia Ursache, Romeo Popa, Mircea Mircean

**Affiliations:** 1 Department of Clinical Sciences, Faculty of Veterinary Medicine, University of Agricultural Sciences and Veterinary Medicine Cluj-Napoca, Calea Mănăştur 3–5, 400372 Cluj-Napoca, Romania; alexandra.biris@usamvcluj.ro (A.B.); daniela.neagu@usamvcluj.ro (D.N.); alina.hasas@usamvcluj.ro (A.D.H.); cristian.popovici@usamvcluj.ro (C.P.); mircea.mircean@usamvcluj.ro (M.M.); 2 Department of Pharmacology, University of Medicine and Pharmacy of Craiova, 200349 Craiova, Romania; romeo_rop@yahoo.com

**Keywords:** cystatin C, N-acetyl-β-D-glucosaminidase, geriatric dogs, renal biomarkers, subclinical kidney disease, tubular injury, prevalence

## Abstract

Serum creatinine (sCr), blood urea nitrogen (BUN) and symmetric dimethylarginine (SDMA) reflect glomerular filtration only and are subject to non-renal confounders including lean body mass, limiting early detection of renal dysfunction in geriatric dogs. We evaluated serum cystatin C (sCysC) and the urinary N-acetyl-β-D-glucosaminidase-to-creatinine ratio (NAG index) as early renal markers in healthy geriatric dogs. Sixty-one neutered geriatric dogs, free of clinical disease and of extrarenal laboratory abnormalities, were stratified into three groups: controls (n = 36), subclinical (normal sCr, ≥1 elevated biomarker; n = 17) and azotaemic chronic kidney disease (CKD; sCr > 1.5 mg/dL; n = 8). sCysC was measured by a canine-specific ELISA and the NAG index by colorimetric assay. Of the 61 dogs, 17 (27.9%) had at least one elevated biomarker with normal sCr, and all 17 also had normal BUN, indicating pre-azotaemic changes identified predominantly by sCysC. The NAG index was elevated in 6/17 (35.3%) subclinical dogs and SDMA in none (0/17). Because sCysC status partly informed group allocation and no independent measure of glomerular filtration rate was obtained, these findings characterise an altered renal biochemical phenotype rather than establish diagnostic accuracy, and require prospective validation.

## Simple Summary

Kidney disease is common in older dogs and is usually recognised late, once a large part of kidney function has already been lost. The blood tests used at routine check-ups become abnormal only after considerable damage has occurred, and in elderly dogs they are further affected by the loss of muscle that comes with age. We examined sixty-one apparently healthy older dogs, measuring two less commonly used markers alongside the standard tests: cystatin C in blood, which reflects how well the kidneys filter waste, and a urinary enzyme that signals injury to the small tubes inside the kidney. More than one in four dogs with a normal routine kidney test had an abnormal result for at least one of these two markers, most often cystatin C. These dogs cannot yet be said to have kidney disease, because kidney filtration was not measured directly, but their laboratory profile differs from that of dogs of the same age with normal results. Adding these two markers to routine health checks in older dogs could help identify animals that deserve closer monitoring, and studies that follow such dogs over time are now needed to establish what these early changes mean.

## 1. Introduction

Chronic kidney disease (CKD) is among the most prevalent conditions in aging dogs, with epidemiological data indicating that dogs older than 12 years have more than five times the odds of developing CKD compared with dogs aged 7 to 12 years [1]. Despite this well-established association between advancing age and renal deterioration, the early detection of kidney dysfunction in geriatric dogs remains a significant clinical challenge, largely because conventional renal biomarkers retain values within the reference interval until glomerular filtration rate (GFR) has declined by approximately 75% [2]. This diagnostic delay implies that, by the time azotemia becomes measurable, the majority of functional renal mass has already been irreversibly lost.

Serum creatinine, the most widely used surrogate marker of GFR in veterinary practice, carries additional limitations that are particularly relevant in the geriatric patient [3]. As a product of muscle metabolism, its serum concentration is directly dependent on lean body mass, and geriatric dogs commonly develop sarcopenia and progressive muscle wasting as part of the normal aging process [4]. As a result, serum creatinine may remain within the reference interval in elderly dogs with significantly compromised renal function, generating a falsely reassuring clinical picture [2]. Blood urea nitrogen (BUN) is similarly unreliable, being influenced by dietary protein intake, hydration status, gastrointestinal haemorrhage, and hepatic function, independently of renal filtration capacity [2,5]. Symmetric dimethylarginine (SDMA), introduced more recently into routine veterinary diagnostics, represents an advance over creatinine in that it detects decreases in GFR at an earlier stage, with serum concentrations reported to rise an average of 9.8 months before serum creatinine in a small group of dogs with progressive CKD sampled at irregular intervals, a lead time that should therefore be regarded as an approximation [2], and it is not influenced by changes in lean body mass [6]. Nevertheless, SDMA, creatinine, and BUN all reflect glomerular filtration exclusively and provide no information about the tubular compartment of the nephron, which may sustain early injury independently and before glomerular function deteriorates [7]. SDMA is also subject to non-renal influences of its own: concentrations are breed-dependent and are higher in Greyhounds than in non-sighthound breeds [8], are frequently increased in dogs with lymphoma in the absence of azotaemia [9], and decrease as body fat percentage rises in otherwise healthy dogs [10].

Two biomarkers that address distinct aspects of renal physiology beyond glomerular filtration have received comparatively limited attention in the context of geriatric canine medicine: N-acetyl-β-D-glucosaminidase (NAG) and cystatin C (CysC). NAG is a lysosomal enzyme of high molecular weight (approximately 140 kDa) present in the epithelial cells of the proximal convoluted tubule. Because of its large molecular size, it is not filtered under physiological conditions by the intact glomerulus; its appearance in urine therefore reflects proximal tubular cell injury or necrosis directly, making urinary NAG activity a sensitive and specific indicator of tubular damage [11,12]. The urinary NAG-to-creatinine ratio (NAG index), which corrects for variations in urine concentration, has been shown to increase in dogs with CKD, pyelonephritis, and other nephropathies [13], and age-related elevation of this ratio has been documented in dogs older than six years even in the absence of overt renal disease [14,15]. Serum cystatin C, by contrast, is a low-molecular-weight cysteine proteinase inhibitor (approximately 13 kDa) produced at a constant rate by all nucleated cells, freely filtered by the glomerulus, and subsequently reabsorbed and catabolised in the proximal tubule without returning to circulation [16]. Its serum concentration is therefore determined almost exclusively by GFR and, critically, is not influenced by lean body mass, age, sex, or dietary protein content in the manner that serum creatinine is [16]. In dogs, serum CysC has been shown to correlate with age in small-breed dogs without azotaemia, suggesting that it may be sensitive to subclinical, aging-associated declines in renal filtration that fall below the detection threshold of creatinine [17,18]. A recent longitudinal study in apparently healthy aging dogs further demonstrated that serum CysC concentrations are associated with the subsequent development of azotaemic CKD and mortality, underscoring its potential prognostic value in geriatric screening [19].

While individual studies have investigated NAG or CysC in dogs with established renal disease or in the context of specific conditions such as leishmaniosis or nephrotoxic drug exposure, the combined evaluation of the NAG index and serum CysC in a population of clinically healthy geriatric dogs without pre-existing renal, hepatic, or haematological abnormalities has not been reported. The absence of reference data specific to this population limits the clinical interpretation of these markers in routine geriatric screening, where the goal is to detect subclinical renal impairment before conventional markers become abnormal.

The present study aimed to characterise serum CysC concentrations and the NAG index in clinically healthy geriatric dogs and to characterise their behaviour in relation to conventional markers, using a three-tier design comprising a healthy control group, a subclinical group with normal serum creatinine, and an azotaemic CKD group. Dogs were classified as geriatric based on body weight, with a threshold of eight years for dogs weighing 15 kg or more and ten years for dogs weighing less than 15 kg, in accordance with established veterinary literature recognising the inverse relationship between body size and rate of ageing in dogs [20,21].

## 2. Materials and Methods

### 2.1. Study Design and Ethical Approval

This cross-sectional observational study was conducted between March and November 2025 and enrolled clinically healthy geriatric dogs presented by their owners at the Clinical Teaching Hospital of the Faculty of Veterinary Medicine, Cluj-Napoca, Romania, and at collaborating veterinary practices in Cluj County and adjacent counties. Recruitment was performed through targeted announcements distributed via social media and directly to collaborating clinics, inviting owners of dogs without known chronic disease and without ongoing medical treatment to contact the research team. A total of 131 dogs were registered, of which 61 met all inclusion criteria and were enrolled in the study.

The study protocol was approved by the Bioethics Committee of the University of Agricultural Sciences and Veterinary Medicine Cluj-Napoca (protocol code 569, date of approval 12 June 2024). Written informed consent was obtained from all owners prior to enrolment, including consent for the publication of study results.

### 2.2. Animals: Inclusion and Exclusion Criteria

Dogs were classified as geriatric based on body weight, with a threshold of eight years for dogs weighing 15 kg or more and ten years for dogs weighing less than 15 kg, in accordance with established veterinary literature recognising the inverse relationship between body size and rate of ageing in dogs [20,21]. Inclusion criteria were: age meeting the geriatric threshold for the respective body weight category; neutered status (male castrated or female spayed); absence of any ongoing pharmacological treatment at the time of enrolment; no history of chronic disease; and normal results on physical examination, haematology, serum biochemistry, and urinalysis as defined below. Serum creatinine and the three novel biomarkers were deliberately excluded from these criteria: dogs were not required to have normal renal analytes, and these variables served instead as the basis for group allocation (Section 2.3). All other haematological, biochemical and urinalysis abnormalities led to exclusion.

Exclusion criteria were applied sequentially. In the first stage, 17 dogs were excluded due to a documented history of chronic disease and 12 due to ongoing pharmacological treatment at the time of the consultation. In the second stage, 11 dogs were excluded based on abnormalities identified at physical examination, including fever, cardiac murmur, systolic arterial hypertension, or ultrasonographic findings suggestive of organic pathology. In the third stage, 30 dogs were excluded due to abnormal haematological, serum biochemical, or urinalysis parameters. A total of 70 dogs were excluded, yielding a final study population of 61 dogs.

### 2.3. Group Allocation

Within the final population of 61 dogs, three groups were defined a priori on the basis of serum creatinine and the status of the novel biomarkers. Each biomarker was classified as elevated when its concentration exceeded the upper reference limit of the corresponding assay: serum cystatin C > 0.86 mg/L, SDMA > 14 µg/dL, and NAG index > 2.5 U/g creatinine. The SDMA threshold corresponds to the established canine reference limit [2,7,22], whereas the serum cystatin C and urinary NAG index thresholds correspond to the reference limits of the canine ELISA and colorimetric assays used (Section 2.6), in line with previously reported canine values [13,16]. The healthy control group comprised dogs with normal serum creatinine (≤1.5 mg/dL) and all three novel biomarkers within reference limits (n = 36). The subclinical group comprised dogs with normal serum creatinine (≤1.5 mg/dL) but at least one elevated novel biomarker (n = 17), representing the population of primary interest for early detection. The azotaemic CKD group comprised dogs with serum creatinine above the reference limit (>1.5 mg/dL), consistent with International Renal Interest Society (IRIS) creatinine ranges suggestive of early-stage CKD, although full IRIS staging criteria were not applied [22], and served as an internal validation group to confirm that the novel biomarkers track established renal impairment. It should be noted that, because control dogs were required to have all novel biomarkers within reference limits and subclinical dogs were required to have at least one elevated novel biomarker, the discrimination of these two groups by any individual biomarker is partially inherent to the allocation criteria; this design consideration is addressed further in the Discussion below.

### 2.4. Clinical Examination

All dogs underwent a standardised clinical assessment performed by three experienced veterinary clinicians. The assessment comprised a complete physical examination, measurement of systolic arterial blood pressure (SAP), and abdominal ultrasonography. Systolic arterial blood pressure was measured by Doppler sphygmomanometry following a minimum acclimatisation period of 30 min, with the mean of four consecutive measurements recorded. Dogs with a mean SAP exceeding 160 mmHg were excluded, in accordance with the American College of Veterinary Internal Medicine (ACVIM) consensus guidelines [23]. Abdominal ultrasonography was performed using an Esaote MyLab X8 unit (Esaote S.p.A., Genoa, Italy) equipped with a microconvex transducer operating at a variable frequency of 3 to 11 MHz. Ultrasonographic examination was used exclusively as a screening tool for structural organ pathology; quantitative measurements were not recorded. Body condition was scored on a nine-point scale and muscle condition was scored separately, using the World Small Animal Veterinary Association muscle condition score graded from 0 (normal muscle mass) to 3 (severe muscle loss), by the same clinician for each dog.

### 2.5. Sample Collection

All blood and urine samples were collected on the same morning as the clinical examination, following a minimum overnight fast of eight hours (12 h recommended), with free access to water throughout. Owners were instructed to collect the first morning urine sample at home by free-catch midstream technique using a sterile urine collection container provided by the research team. Samples were transported to the laboratory and processed within a maximum of two hours from collection. Urine specific gravity was measured by refractometry immediately upon receipt. Urinary biochemistry was assessed by photometric reading of a reagent dipstick. Urine sediment was evaluated by light microscopy following centrifugation at 1800 rpm (400× *g*) for five minutes at room temperature. Aliquots of the supernatant were stored at −20 °C until batch analysis.

Blood was collected by venepuncture of the cephalic vein into three separate tubes: a plain serum tube for serum biochemistry and serum cystatin C (CysC) determination, a lithium heparin tube for SDMA analysis, and an EDTA tube for complete blood count. Blood collection was performed before 12:00. Serum tubes were allowed to clot for ten minutes and then centrifuged at 4000 rpm (1900× *g*); serum was separated within a maximum of 20 to 30 min. One serum aliquot was used for same-day biochemistry analysis; a second aliquot and the plasma fraction from the lithium heparin tube were stored at −20 °C until batch analysis of CysC and SDMA, respectively. The EDTA tube was analysed within one hour of collection at room temperature.

### 2.6. Laboratory Analyses

All analyses were performed at the Clinical Laboratory of the Faculty of Veterinary Medicine Cluj-Napoca by an experienced biochemist. Complete blood count was performed using a 5-differential haematology analyser (LaserCyte Dx, IDEXX Laboratories, Inc., Westbrook, ME, USA). Serum biochemistry, including blood urea nitrogen (BUN), alanine aminotransferase (ALT), alkaline phosphatase (ALP), gamma-glutamyltransferase (GGT), total bilirubin, total protein, albumin, glucose, sodium, potassium, chloride, calcium, and phosphorus, was determined on an IDEXX Catalyst One analyser (IDEXX Laboratories, Inc., Westbrook, ME, USA). Serum creatinine was measured separately by the kinetic Jaffe method on a Hospitex Screen Master Touch spectrophotometer (Hospitex Diagnostics, Sesto Fiorentino, Italy). SDMA was measured using the IDEXX SDMA immunoassay on an IDEXX Catalyst One analyser (IDEXX Laboratories, Inc., Westbrook, ME, USA). Reference intervals used for the assessment of haematological and biochemical parameters were those established by Villiers and Ristic [24] and Weiss and Wardrop [25].

Serum CysC concentration was determined by enzyme-linked immunosorbent assay (ELISA) using a commercially available canine-specific cystatin C ELISA kit (Canine Cystatin C ELISA, catalogue number BA2008; BioLab Assays, Brno, Czech Republic), a solid-phase sandwich immunoassay calibrated against a recombinant canine cystatin C standard (calibration range 0.31–10 ng/mL). Serum samples were assayed using the manufacturer’s recommended two-step dilution procedure (final dilution 1:1000), and concentrations were multiplied by the dilution factor and expressed in mg/L. The manufacturer reports a limit of detection below 0.005 ng/mL, intra-assay and inter-assay coefficients of variation of 4.2–8.4% and 4.1–7.3%, respectively, dilution linearity of 86–96% and spiking recovery of 98–108%. Absorbance was read at 450 nm on a Biosan HiPo MPP-96 microplate photometer (Biosan Ltd., Riga, Latvia), with incubation performed on a Biosan PST-60HL thermo-shaker (Biosan Ltd., Riga, Latvia). ELISA was preferred over immunoturbidimetric methods, which require species-specific validation in dogs [26]. Urinary NAG activity was measured by colorimetric assay on a Hospitex Screen Master Touch spectrophotometer (Hospitex Diagnostics, Sesto Fiorentino, Italy) using a commercially available N-acetyl-β-D-glucosaminidase assay kit (catalogue number DZ062A; Diazyme Laboratories, Inc., Poway, CA, USA). Urinary creatinine concentration was determined on the same analyser by the kinetic Jaffe method. The NAG index was calculated by dividing urinary NAG activity (expressed in U/L) by urinary creatinine concentration (expressed in g/L), yielding a result in U/g creatinine, thereby correcting for variations in urine concentration.

### 2.7. Statistical Analysis

Statistical analyses were performed in Python 3.12 (Python Software Foundation, Beaverton, OR, USA), scikit-learn, pandas, pingouin and scikit-posthocs libraries. The normality of distribution of all continuous variables was assessed using the Shapiro–Wilk test. Normally distributed continuous variables are presented as mean ± standard deviation (SD), while non-normally distributed variables are expressed as median with interquartile range [Q1–Q3]. Categorical variables are reported as absolute frequencies and percentages. Comparisons among the three groups were performed using the Kruskal–Wallis test for non-normally distributed variables or one-way analysis of variance for normally distributed variables, with the Mann–Whitney U test (with effect size r = Z/√N) or the independent-samples *t*-test following Levene’s test (with Cohen’s d) for pairwise comparisons. Post hoc pairwise testing following a significant Kruskal–Wallis result was performed with Dunn’s test and Bonferroni adjustment. Because Dunn’s test uses ranks pooled across all three groups, its results are not numerically identical to the pre-specified Mann–Whitney comparisons restricted to the subclinical and control groups. Categorical variables were compared using Pearson’s chi-square test or Fisher’s exact test when expected cell counts were below five. A Bonferroni correction was applied for the three primary biomarker comparisons (serum cystatin C, SDMA and NAG index), yielding an adjusted significance threshold of *p* < 0.017. Correlations between biomarkers were assessed using Spearman’s rank correlation coefficient. A principal component analysis (PCA) was performed on standardised data to explore multivariate separation between groups. Statistical significance was set at *p* < 0.05 unless otherwise specified. During the preparation of this manuscript the authors used Claude (Anthropic, San Francisco, CA, USA) for language editing and manuscript formatting; it was not used for study design, data collection, statistical analysis or interpretation of results.

## 3. Results

### 3.1. Study Population and Group Allocation

**Figure 1:**
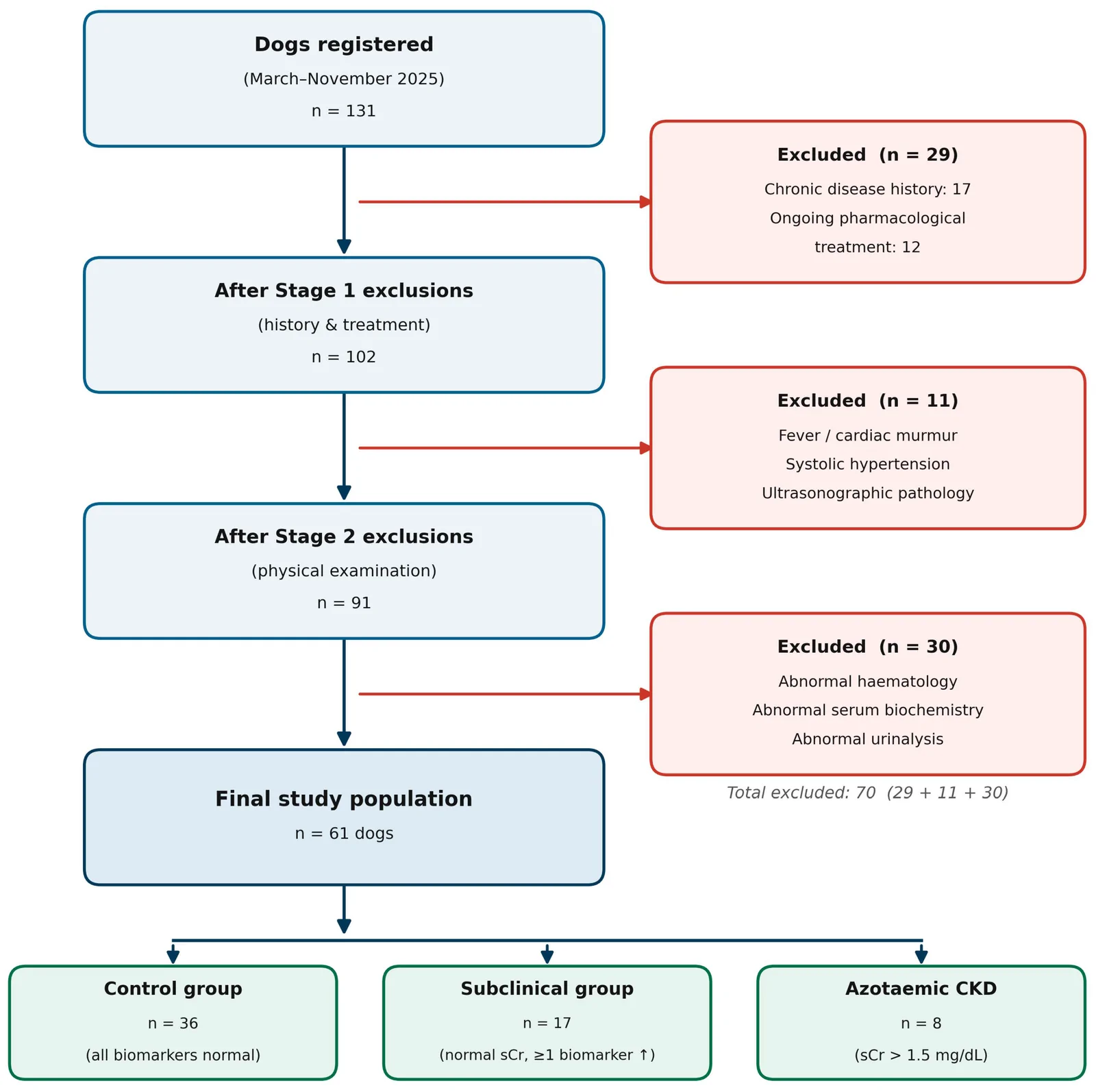
Flow chart of study recruitment and group allocation. Dogs were excluded sequentially across three stages: history and ongoing treatment (n = 29; chronic disease history, 17; ongoing pharmacological treatment, 12), physical examination (n = 11), and laboratory parameters (n = 30; abnormal haematology, serum biochemistry, or urinalysis). The final population of 61 dogs was stratified into three groups on the basis of serum creatinine and novel biomarker status: control (n = 36), subclinical (n = 17), and azotaemic CKD (n = 8).

Of the 131 dogs initially registered, 61 met all inclusion criteria and were enrolled. Group allocation yielded 36 dogs in the healthy control group, 17 dogs in the subclinical group, and 8 dogs in the azotaemic CKD group. In the subclinical group, serum cystatin C was the most frequently elevated biomarker, present in 16 of 17 dogs (94.1%), followed by the NAG index in 6 of 17 dogs (35.3%); SDMA was not elevated in any subclinical dog (0 of 17). In the azotaemic CKD group, all 8 dogs had elevated SDMA, while serum cystatin C and the NAG index were elevated in 4 and 5 dogs, respectively. By design, all biomarkers were within reference limits in every control dog. The three groups did not differ significantly in age (*p* = 0.699), body weight (*p* = 0.347), body condition score (*p* = 0.535), sex distribution (*p* = 0.742) or breed composition (*p* = 0.269), confirming demographic homogeneity across groups. The complete recruitment flow is illustrated in Figure 1.

### 3.2. Descriptive Statistics

Descriptive statistics for all groups are presented in Table 1. Serum cystatin C showed a statistically significant difference across groups, with a median of 1.05 [0.93–1.16] mg/L in the subclinical group, 0.70 [0.50–0.77] mg/L in controls, and 0.86 [0.65–1.38] mg/L in azotaemic CKD dogs (*p* < 0.001). SDMA showed the highest observed values in the azotaemic group (n = 8; interpreted with caution given the small sample size) (28.65 [23.00–30.95] µg/dL) but only modest elevation in the subclinical group (7.90 [6.60–9.30] µg/dL) relative to controls (5.00 [4.07–7.88] µg/dL). The NAG index followed a broadly comparable pattern, increasing from 0.88 [0.42–1.17] U/g in controls to 1.39 [0.58–2.64] U/g in subclinical dogs and 3.67 [1.45–4.20] U/g in azotaemic dogs (*p* = 0.005). As expected from the group definitions, serum creatinine and BUN were highest in the azotaemic group, while urine protein-to-creatinine ratio, urine specific gravity and the hepatic and haematological parameters did not differ significantly among groups, confirming the absence of confounding extrarenal disease. Post hoc testing (Dunn’s test with Bonferroni adjustment) showed that serum cystatin C differed between subclinical dogs and controls (*p* < 0.001) and between azotaemic CKD dogs and controls (*p* = 0.030), but not between subclinical and azotaemic CKD dogs (*p* = 0.369). SDMA differed between azotaemic CKD dogs and both controls (*p* < 0.001) and subclinical dogs (*p* = 0.008), but not between subclinical dogs and controls (*p* = 0.095). The NAG index differed only between azotaemic CKD dogs and controls (*p* = 0.008). The median serum cystatin C recorded in the azotaemic CKD group is lower than that of the subclinical group; this follows from the group definitions and is addressed in Section 4.

### 3.3. Between-Group Comparisons of Renal Biomarkers

Pairwise comparison between the subclinical and control groups (Table 2) revealed a highly significant difference in serum cystatin C (Mann–Whitney U = 612, *p* < 0.001), with a very large effect size (r = 0.805), which remained significant after Bonferroni correction (adjusted threshold *p* < 0.017). SDMA also differed between subclinical dogs and controls (U = 436, *p* = 0.014, r = 0.338), surviving Bonferroni correction, although the effect size was moderate and every value in both groups lay within the reference interval (subclinical dogs, 4.1–11.8 µg/dL; controls, maximum 11.6 µg/dL). This difference is therefore statistically detectable at group level but has no diagnostic application in the individual dog. The NAG index did not differ significantly between the two groups once the Bonferroni-adjusted threshold was applied (U = 414, *p* = 0.041, r = 0.281), and the associated effect size was small. Systolic arterial pressure was significantly higher in subclinical dogs than in controls (U = 556, *p* < 0.001, r = 0.652). Serum creatinine differed only modestly between the two groups (*p* = 0.044), whereas BUN did not differ significantly (*p* = 0.076), and the urine protein-to-creatinine ratio did not differ (*p* = 0.782).

**Table 2:** Pairwise comparison between the subclinical group (n = 17) and the healthy control group (n = 36). Effect size r was calculated as Z/√N. † indicates a comparison that remained significant after Bonferroni correction for the three primary biomarkers (adjusted threshold *p* < 0.017).

Parameter	Test	Statistic	*p*-Value	Effect Size
Serum cystatin C	Mann–Whitney U	U = 612	<0.001 †	r = 0.805
SDMA	Mann–Whitney U	U = 436	0.014 †	r = 0.338
Urinary NAG index	Mann–Whitney U	U = 414	0.041	r = 0.281
Serum creatinine	Mann–Whitney U	U = 412	0.044	r = 0.277
BUN	Mann–Whitney U	U = 400	0.076	r = 0.243
UPC	Mann–Whitney U	U = 321	0.782	r = 0.038
Systolic arterial pressure	Mann–Whitney U	U = 556	<0.001	r = 0.652

### 3.4. Biomarker Elevation Beyond Creatinine

Among all 61 enrolled dogs, 53 had a normal serum creatinine (≤1.5 mg/dL). Within this normocreatinaemic population, 17 dogs (27.9% of the total cohort) had at least one elevated novel biomarker. All 17 of these dogs also had blood urea nitrogen within the reference interval (7–27 mg/dL), confirming genuinely pre-azotaemic subclinical renal changes. In a sensitivity analysis, varying the serum cystatin C cut-off by ±10% shifted this prevalence estimate from 19.7% (12/61) to 42.6% (26/61), indicating its dependence on the chosen threshold. In this pre-azotaemic subset, serum cystatin C was elevated in 16 of the 17 dogs and the NAG index in 6; SDMA was elevated in none.

### 3.5. Biomarker Score and the Renal Impairment Gradient

A composite biomarker score (0–3), reflecting the number of elevated novel biomarkers, increased progressively across the disease spectrum. In the subclinical group, 12 dogs had a score of 1 and 5 dogs a score of 2, with no subclinical dog reaching a score of 3, whereas in the azotaemic CKD group the score was more evenly distributed towards higher values (2 dogs with score 1, 3 dogs with score 2, and 3 dogs with score 3); notably, no dog in the azotaemic CKD group had a biomarker score of 0, confirming that every azotaemic dog had at least one elevated novel biomarker. The biomarker score correlated significantly with systolic arterial pressure (ρ = 0.654, *p* < 0.001), SDMA (ρ = 0.556, *p* < 0.001), serum creatinine (ρ = 0.491, *p* < 0.001) and BUN (ρ = 0.479, *p* < 0.001), but not with age (ρ = −0.041, *p* = 0.756). Kruskal–Wallis testing confirmed a significant gradient in serum creatinine (H = 16.81, *p* < 0.001), BUN (H = 15.08, *p* = 0.002) and systolic arterial pressure (H = 27.10, *p* < 0.001) across score categories, supporting the biological coherence of the composite score as a marker of cumulative renal burden.

### 3.6. Correlations Among Renal Biomarkers and Clinical Variables

**Figure 2:**
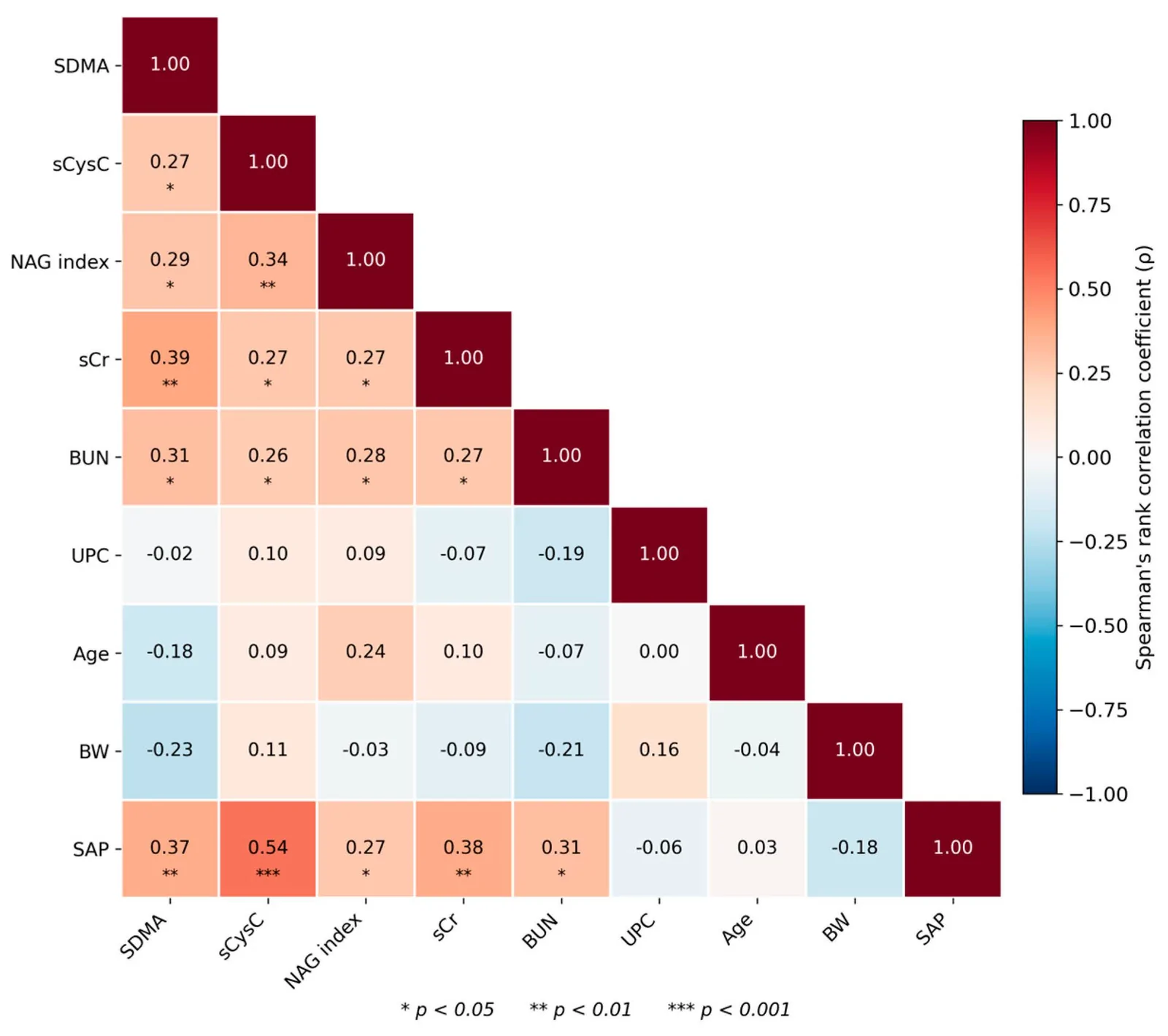
Spearman correlation matrix of renal biomarkers and clinical variables in all 61 dogs. Colour intensity reflects the strength and direction of the correlation coefficient (ρ). Asterisks denote statistical significance (* *p* < 0.05; ** *p* < 0.01; *** *p* < 0.001). sCysC, serum cystatin C; NAG index, urinary N-acetyl-β-D-glucosaminidase-to-creatinine ratio; sCr, serum creatinine; BUN, blood urea nitrogen; UPC, urine protein-to-creatinine ratio; BW, body weight; SAP, systolic arterial pressure.

Spearman correlations among renal biomarkers and clinical variables across all 61 dogs are presented in Figure 2. The strongest association was observed between serum cystatin C and systolic arterial pressure (ρ = 0.543, *p* < 0.001). SDMA correlated moderately with serum creatinine (ρ = 0.392, *p* = 0.002), consistent with both being glomerular markers. Serum cystatin C correlated significantly with the NAG index (ρ = 0.340, *p* = 0.007), suggesting partial overlap between glomerular and tubular involvement in subclinical disease. Notably, none of the novel biomarkers correlated significantly with age or body weight, indicating that the observed elevations reflect renal status rather than the confounding effects of body size or ageing per se.

### 3.7. Multivariate Separation by Principal Component Analysis

**Figure 3:**
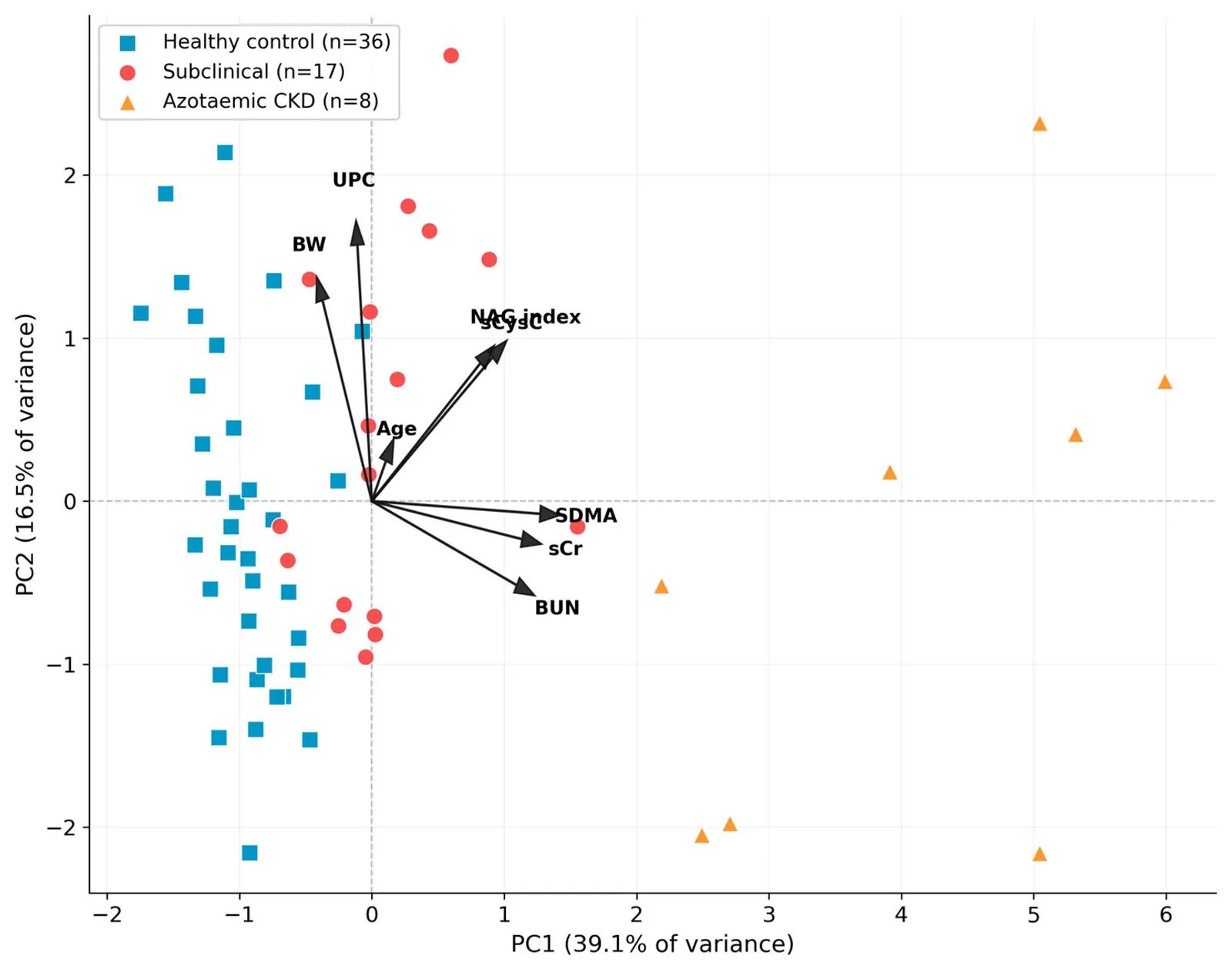
Principal component analysis (PCA) biplot of renal biomarkers in geriatric dogs (n = 61). Each point represents an individual dog, coloured by group. Arrows indicate the loadings of the original variables on the first two principal components. Healthy controls cluster on the left, azotaemic CKD dogs on the right, and subclinical dogs occupy an intermediate position, consistent with a continuum of renal impairment. One subclinical dog positioned apart from the remainder of the subclinical cluster combined the highest urinary NAG index recorded in the subclinical group with a concurrently elevated serum cystatin C; because the second component was driven principally by the urine protein-to-creatinine ratio and body weight, this position reflects the heterogeneous biomarker and body-size patterns captured within the group rather than an isolated marker elevation.

Principal component analysis of the standardised biomarker data (Figure 3) demonstrated clear multivariate separation among the three groups. The first two principal components together explained 55.6% of the total variance (PC1 39.1%, PC2 16.5%). The first component, dominated by SDMA, serum creatinine and BUN, separated azotaemic CKD dogs from the remainder of the cohort along a gradient of glomerular dysfunction, while the subclinical dogs occupied an intermediate position between healthy controls and azotaemic dogs. This ordination is consistent with the conceptualisation of subclinical renal change as an intermediate stage on a continuum between renal health and overt azotaemic CKD. Because the groups were defined using the same variables that entered the analysis, the separation shown here illustrates the magnitude of the differences in biochemical profile between groups but does not constitute independent validation of the group structure.

### 3.8. Comparison Between Included and Excluded Dogs

To assess the potential for selection bias, the 61 included dogs were compared with the 41 dogs excluded during the clinical and laboratory screening stages (Stages 2 and 3). Dogs excluded at Stage 1 (n = 29) were screened by telephone history prior to clinical presentation and did not have demographic data recorded in a format available for comparison. No significant differences were found in age (*p* = 0.540), sex distribution (*p* = 0.419) or breed composition (*p* = 0.937), indicating that the enrolled population was demographically representative of the screened geriatric dogs. Among the dogs excluded at the clinical examination stage (n = 11), the most frequent reasons were systolic hypertension and cardiac murmur (5 dogs each), with a median systolic arterial pressure of 168 mmHg [IQR 165–170] among those excluded for hypertension. Among the 30 dogs excluded for laboratory abnormalities, 21 were excluded on the basis of serum biochemistry, 3 on urinalysis and 6 on both.

## 4. Discussion

This study provides the first combined evaluation of serum cystatin C and the urinary NAG index in a well-characterised population of clinically healthy geriatric dogs with normal serum creatinine. The central finding is that more than one in four apparently healthy geriatric dogs (27.9%) showed an altered renal biochemical profile that conventional glomerular markers did not identify in this cohort, and that serum cystatin C accounted for almost all of these animals. These results have direct implications for how renal screening is approached in the ageing canine patient.

Serum cystatin C was the biomarker most frequently elevated in the subclinical group. Its value as a filtration marker is biologically plausible, since cystatin C is produced at a constant rate by all nucleated cells, is freely filtered by the glomerulus and, unlike creatinine, is independent of lean body mass [6,16]. In a geriatric population in which sarcopenia is common and creatinine is correspondingly unreliable [4,27], a filtration marker that is unaffected by muscle mass is in principle valuable, although body and muscle condition scores did not differ between the groups of the present cohort. Cystatin C has been shown to track glomerular filtration rate in dogs across body sizes [28] and to perform comparably to or better than creatinine and SDMA for the detection of reduced GFR [29], although in the present cohort it was less consistently increased than SDMA in the azotaemic group, a discrepancy addressed below. Our findings are consistent with earlier reports that serum cystatin C correlates with age-associated declines in filtration in non-azotaemic dogs [17], is predictive of subsequent azotaemic CKD and mortality in apparently healthy aging dogs [19], and identifies renal involvement in systemic conditions such as diabetes mellitus and hyperadrenocorticism [30,31]. Importantly, dogs with persistently elevated cystatin C in the absence of clinical signs warrant monitoring rather than dismissal [32]. The present data are consistent with these observations, but the separation of the subclinical and control groups by cystatin C follows in part from the allocation criteria and cannot be read as independent evidence of diagnostic accuracy.

The urinary NAG index occupied a complementary but secondary role. It exceeded the assay reference limit of 2.5 U/g creatinine in 6 of the 17 subclinical dogs (35.3%), compared with no control dog, and correlated with serum cystatin C, suggesting that a subset of dogs with early glomerular involvement also sustain proximal tubular injury. However, its overall discriminatory performance was modest and statistically indistinguishable from that of serum creatinine. This is in keeping with the known biology of NAG as a marker of active tubular damage rather than of filtration capacity [11,12], and with reports that the NAG index rises in established nephropathies, in urinary tract disease, and with age [13,14]. Reference indices for urinary NAG activity in clinically normal dogs have been established [33], and combined urinary cystatin C and NAG measurement has shown value as an early marker in canine renal disease of diverse aetiology, including leishmaniosis [34]. Urinary cystatin C has likewise been reported to increase across CKD stages [35]. The NAG index may therefore be most useful not as a standalone screening test but as an adjunct that identifies the tubular component of renal injury in dogs already flagged by an abnormal filtration marker.

Perhaps the most clinically instructive finding concerns SDMA. Although SDMA is widely promoted as an early marker of GFR decline [36,37] and performed strongly in our azotaemic CKD group, where every dog had an elevated value, it identified none of the subclinical dogs (0 of 17). In other words, in dogs with genuinely normal creatinine, SDMA failed to flag any of the early impairment that cystatin C readily detected. This dissociation suggests that SDMA and cystatin C, despite both being filtration markers, may capture different points on the trajectory of declining renal function, with cystatin C sensitive to earlier or more subtle reductions in filtration. The interpretation of SDMA in older dogs is further complicated by the influence of the analytical method and by age-related shifts in its reference interval [38]. This observation argues against reliance on SDMA alone for the earliest detection of renal impairment in geriatric dogs and supports the complementary use of cystatin C, consistent with the comparative findings of Pelander et al. [29], who reported that cystatin C and SDMA performed similarly for the detection of reduced GFR but that cystatin C may be more sensitive at earlier stages of dysfunction.

The contrast between sCysC and SDMA in the subclinical group must be read in the light of where the two decision thresholds fall relative to the distributions observed in this population. The upper reference limit of the canine ELISA used for sCysC, 0.86 mg/L, lies close to the upper part of the control distribution (median 0.70 mg/L, third quartile 0.77 mg/L), so that a modest shift in filtration is sufficient to move an animal above it. The SDMA threshold of 14 µg/dL lies well beyond the values observed in either non-azotaemic group, in which no dog exceeded 11.8 µg/dL. A marker whose cut-off sits at the margin of the healthy distribution will classify more animals as abnormal than a marker whose cut-off sits far above it, irrespective of the underlying physiology. The apparent advantage of sCysC over SDMA in the pre-azotaemic range therefore reflects, at least in part, the relative position of the two thresholds and the analytical characteristics of the respective assays, rather than a demonstrated physiological difference in sensitivity. The dependence of the prevalence estimate on the sCysC cut-off reported in Section 3.4 makes the same point. A fair comparison of the two markers would require both to be referenced to a common measure of glomerular filtration rate in the same animals.

The absence of proteinuria in the subclinical group merits comment. The urine protein-to-creatinine ratio did not differ across groups and remained within reference limits in every dog, including those with elevated cystatin C. Rather than contradicting the presence of early renal impairment, this pattern is consistent with it: proteinuria typically emerges with glomerular barrier dysfunction or more advanced tubulointerstitial disease, whereas the changes detected here by cystatin C reflect an early, predominantly filtration-level deficit that precedes overt protein loss. The preservation of normal UPC therefore supports the interpretation that the subclinical dogs occupy a genuinely early, pre-azotaemic and pre-proteinuric stage of renal impairment, and it reinforces the value of a sensitive filtration marker such as cystatin C for detecting renal change before conventional indicators of glomerular or tubular damage become abnormal.

The association between serum cystatin C and systolic arterial pressure, which was the strongest correlation observed in this cohort, deserves further attention. Sustained systemic hypertension causes afferent arteriolar vasoconstriction and a progressive reduction in effective filtration pressure, leading to a measurable decrease in GFR that is reflected in rising cystatin C before creatinine crosses its threshold. This raises the possibility that a subset of the subclinical dogs had early hypertensive nephropathy rather than, or in addition to, age-related nephron loss, and suggests that cystatin C may serve as a combined marker of renal and cardiovascular risk in the geriatric dog. A consequent limitation is that the present study cannot distinguish between cystatin C elevation of intrinsic renal origin, reflecting true nephron loss or tubulo-glomerular damage, and elevation of haemodynamic origin secondary to systemic hypertension; the label ‘subclinical renal impairment’ may therefore encompass a heterogeneous group. Differentiating these mechanisms would require ambulatory blood pressure monitoring and longitudinal follow-up, which future studies should incorporate alongside renal biomarkers.

The three-tier structure of our cohort, spanning healthy controls, subclinical dogs and azotaemic CKD dogs, allowed the renal impairment continuum to be examined directly. The progressive increase in the composite biomarker score across groups, its significant correlations with creatinine, BUN and systolic arterial pressure, and the ordered separation of the three groups in principal component space all support the interpretation of subclinical impairment as an intermediate stage between renal health and overt azotaemic disease. A similar layering of renal biomarkers across disease stages has been described for other markers and in other species, including the cat, where panels of glomerular and tubular markers are increasingly advocated for early detection [39]. The consistent association between renal biomarkers and systolic arterial pressure across analyses is noteworthy and aligns with the well-recognised bidirectional relationship between hypertension and renal disease in dogs [23], and with observations that renal function markers change in parallel with systemic endocrine and haemodynamic status [40], although the cross-sectional design precludes inference about directionality.

An apparently counterintuitive finding warrants explicit comment: the median sCysC recorded in the azotaemic CKD group (0.86 mg/L) was lower than that of the subclinical group (1.05 mg/L). Three considerations account for this. First, the two medians are not statistically distinguishable (Dunn’s test, *p* = 0.369), whereas sCysC in the azotaemic group was significantly higher than in controls (*p* = 0.030) and exceeded the cut-off in four of the eight dogs; sCysC therefore did track azotaemic disease, but less consistently than SDMA. Second, the subclinical group was defined in part by an elevated sCysC, so its distribution is truncated from below at the cut-off and its median is an upwardly selected quantity, whereas the azotaemic group, defined by sCr alone, was subject to no such selection; the two medians are consequently not comparable. Third, an explanation based on differences in muscle mass is not supported by the present data, since neither body condition score (*p* = 0.535) nor muscle condition score (*p* = 0.576) differed across groups. What remains is the combination of a small azotaemic group (n = 8), the wide biological variability of canine cystatin C, which limits the performance of population-based reference limits in the individual animal [17], and the demonstrated sensitivity of classification to the chosen cut-off (Section 3.4). Distinguishing between these contributions would require concurrent measurement of glomerular filtration rate, which the present design did not include.

This study has several limitations that qualify its interpretation. First, renal status was defined by the index biomarkers themselves rather than by an independent reference measure of glomerular filtration rate, such as iohexol clearance; the classification of subclinical renal change is therefore biochemical and hypothesis-generating rather than a demonstrated filtration deficit. Second, the cross-sectional design cannot establish that the affected dogs are progressing towards azotaemic disease; the descriptor pre-azotaemic denotes only the concurrent finding of normal serum creatinine and BUN, and longitudinal follow-up is required to confirm progression. Third, serum cystatin C was measured with a commercial canine ELISA, relying on the manufacturer’s reported analytical performance; laboratory-specific reference ranges and independent re-validation were not established, and the estimated prevalence was sensitive to the chosen cut-off (Section 3.4). The non-monotonic pattern of cystatin C across groups, with a higher median in the subclinical than in the small azotaemic group, largely reflects the group definitions, since the subclinical group was defined in part by elevated cystatin C whereas the azotaemic group was defined by creatinine alone. Fourth, the strong association between the novel biomarkers and systolic arterial pressure raises the possibility that some elevations reflect early hypertensive change rather than intrinsic chronic kidney disease. Serum cystatin C correlated more strongly with systolic arterial pressure (ρ = 0.543) than with any other variable measured, and systolic pressure in the subclinical group did not differ from that of the azotaemic group (*p* = 1.000) while being markedly higher than in controls. A haemodynamic contribution to the observed sCysC elevations therefore cannot be excluded, and the subclinical group should be regarded as biochemically rather than aetiologically defined. Fifth, comparisons beyond the three primary biomarkers were not corrected for multiplicity and should be regarded as exploratory. The azotaemic group was itself heterogeneous: it was defined by serum creatinine alone, full IRIS staging was not applied, and four of the eight dogs retained a urine specific gravity of 1.030 or above. It is therefore best regarded as an internal comparison group rather than as a fully characterised CKD population. Finally, recruitment relied on owner-volunteered convenience sampling, and biomarkers were assayed from aliquots stored at −20 °C with urine collected at home; the prevalence estimates therefore apply to a screening population and may be affected by selection and pre-analytical factors.

Notwithstanding these limitations, a provisional practical implication can be drawn. In geriatric dogs presenting for routine health screening with normal creatinine, sCysC identified an altered renal biochemical profile in a substantial proportion of animals in which conventional markers did not, and the NAG index identified a concurrent tubular component in a subset of them. Whether these animals benefit from earlier monitoring or from renoprotective management is not established by a cross-sectional study and requires prospective evaluation against a reference measure of glomerular filtration rate together with longitudinal follow-up.

## 5. Conclusions

In clinically healthy geriatric dogs with normal serum creatinine, more than one in four (27.9%) showed an altered renal biochemical profile that conventional glomerular markers did not identify in this cohort. Serum cystatin C accounted for almost all of these animals and the urinary NAG index identified a subset with concurrent tubular involvement. Because group allocation was partly informed by cystatin C status, and because no independent measure of glomerular filtration rate was obtained, these findings characterise a biochemical phenotype rather than demonstrate diagnostic accuracy. The robust observation is the high prevalence of pre-azotaemic biochemical abnormality in a geriatric screening population. Prospective studies combining sCysC and the NAG index with measured glomerular filtration rate and with blood pressure monitoring are required to establish whether these changes reflect intrinsic nephron loss, a haemodynamic effect of early hypertension, or both, and whether they predict progression to azotaemic disease.

## Figures and Tables

**Table 1:** Descriptive statistics of demographic, biomarker, conventional renal and clinical parameters across the three study groups. Non-normally distributed continuous variables are presented as median [Q1–Q3], normally distributed variables as mean ± SD, and categorical variables as n (%). *p*-values were obtained by Kruskal–Wallis test, one-way ANOVA, or chi-square test as appropriate.

Parameter	Subclinical (n = 17)	Control (n = 36)	CKD (n = 8)	*p*-Value
Demographics				
Age (years)	11.00 [9.00–12.00]	11.00 [9.75–14.00]	11.50 [9.00–14.00]	0.699
Body weight (kg)	28.66 ± 13.76	32.13 ± 16.04	23.48 ± 18.31	0.347
BCS (1–9)	5.00 [4.00–5.00]	5.00 [4.00–6.00]	5.00 [4.75–5.25]	0.535
MCS (0–3)	1.00 [0.00–2.00]	1.00 [0.00–2.00]	1.50 [0.75–2.00]	0.576
Sex (female spayed), n (%)	9 (52.9)	16 (44.4)	3 (37.5)	0.742
Breed (mixed), n (%)	10 (58.8)	19 (52.8)	2 (25.0)	0.269
Renal biomarkers of interest				
Serum cystatin C (mg/L)	1.05 [0.93–1.16]	0.70 [0.50–0.77]	0.86 [0.65–1.38]	<0.001
SDMA (µg/dL)	7.90 [6.60–9.30]	5.00 [4.07–7.88]	28.65 [23.00–30.95]	<0.001
Urinary NAG index (U/g Cr)	1.39 [0.58–2.64]	0.88 [0.42–1.17]	3.67 [1.45–4.20]	0.005
Conventional renal markers				
Serum creatinine (mg/dL)	1.00 [0.80–1.20]	0.80 [0.60–1.02]	2.05 [1.80–2.58]	<0.001
BUN (mg/dL)	17.90 [15.80–23.70]	17.55 [13.05–21.73]	50.30 [41.27–60.73]	<0.001
UPC	0.09 ± 0.04	0.09 ± 0.05	0.08 ± 0.04	0.719
USG	1.033 [1.029–1.036]	1.035 [1.029–1.037]	1.030 [1.028–1.032]	0.154
Urine pH	5.90 [5.70–6.20]	6.10 [5.80–6.50]	6.35 [5.85–6.50]	0.224
Other clinical parameters				
Systolic arterial pressure (mmHg)	148.00 [145.25–150.25]	132.62 [130.44–137.75]	148.12 [144.50–153.38]	<0.001
ALT (U/L)	67.84 ± 24.21	55.32 ± 18.71	72.44 ± 23.76	0.040
ALP (U/L)	103.24 ± 38.76	90.47 ± 36.90	115.88 ± 32.66	0.167
Haematocrit (%)	47.00 [42.20–48.20]	44.85 [42.20–49.65]	42.65 [39.50–43.60]	0.262
Albumin (g/dL)	3.10 ± 0.31	3.19 ± 0.30	3.16 ± 0.26	0.635
Total protein (g/dL)	6.47 ± 0.48	6.70 ± 0.54	6.67 ± 0.41	0.297

## Data Availability

The original contributions presented in this study are included in the article/Supplementary Material. Further inquiries can be directed to the corresponding author(s).

## Supplementary Materials

The following supporting information can be downloaded at https://www.mdpi.com/article/10.3390/vetsci13090881/s1, Table S1, anonymised clinical and laboratory dataset of the 61 included dogs; Table S2, anonymised clinical and laboratory data of the excluded dogs.

## Abbreviations

The following abbreviations are used in this manuscript:

ACVIM	American College of Veterinary Internal Medicine
ALP	Alkaline phosphatase
ALT	Alanine aminotransferase
ANOVA	Analysis of variance
BCS	Body condition score
BUN	Blood urea nitrogen
BW	Body weight
CKD	Chronic kidney disease
CysC	Cystatin C
EDTA	Ethylenediaminetetraacetic acid
ELISA	Enzyme-linked immunosorbent assay
GFR	Glomerular filtration rate
GGT	Gamma-glutamyltransferase
IQR	Interquartile range
IRIS	International Renal Interest Society
MCS	Muscle condition score
NAG	N-acetyl-β-D-glucosaminidase
PCA	Principal component analysis
SAP	Systolic arterial pressure
sCr	Serum creatinine
sCysC	Serum cystatin C
SD	Standard deviation
SDMA	Symmetric dimethylarginine
UPC	Urine protein-to-creatinine ratio
USG	Urine specific gravity
WSAVA	World Small Animal Veterinary Association
